# Dynamics of small RNAs in a red-fruited wine grape cultivar infected with Grapevine red blotch virus

**DOI:** 10.1186/s12864-025-11539-4

**Published:** 2025-04-29

**Authors:** Noah Ault, Shuchao Ren, David Payne, Yongfang Li, Asha Srinivasan, Yun Zheng, Ramanjulu Sunkar, Rayapati A. Naidu

**Affiliations:** 1 Department of Plant Pathology, Washington State University - Irrigated Agriculture Research and Extension Center, Prosser, WA 99350 USA; 2 College of Horticulture and Landscape, Yunnan Agricultural University, Kunming, China; 3 Department of Biochemistry & Molecular Biology, Oklahoma State University, Stillwater, OK 74078 USA; 4 Department of Molecular Biology and Biotechnology, College of Agriculture, Vellayani, Thiruvananthapuram, Kerala India

**Keywords:** Grapevine, Vitis vinifera, Grapevine red blotch virus, Geminiviridae, Small RNA, microRNA, microRNA Target, High Throughput Sequencing

## Abstract

**Background:**

Red blotch disease, caused by Grapevine red blotch virus (GRBV, genus *Grablovirus*, family *Geminiviridae*), negatively impacts vine health, fruit yield, and quality, leading to substantial economic losses to growers. While recent studies have enhanced our understanding of the epidemiology of GRBV and its effects, little is known about the molecular basis of the host-virus interactions. Since small RNAs (sRNAs) are known to play a central role in host-virus interactions, this study was undertaken to investigate sRNA dynamics in leaves and berries at two phenological stages (asymptomatic pre- and symptomatic post-véraison) of GRBV-infected grapevines (*Vitis vinifera* cv. Merlot).

**Results:**

Among the 140 microRNAs (miRNAs) detected, 41 isoforms belonging to 18 miRNA families exhibited significant differential expression in response to GRBV infection. Furthermore, 50 miRNAs showed differential expression in samples from pre- and post-véraison stages. A total of 58 conserved and 41 novel targets for known *V. vinifera* miRNAs were validated using degradome sequencing data from leaf samples of pre- and post-véraison stages. Additionally, virus-derived siRNAs (vsiRNAs) specific to GRBV were present only in GRBV-positive samples. The vsiRNAs predominantly ranged from 19 to 24 nucleotides (nt), with the 21nt size being the most abundant. Mapping vsiRNAs across the GRBV genome revealed an uneven distribution, with vsiRNA-generating hotspots predominantly located in the V3 ORF. Of the 83 most abundant vsiRNAs, grapevine target transcripts were identified for eight of them.

**Conclusions:**

Identification of differentially expressed miRNAs and vsiRNAs, as well as their targets, offered important insights into various pathways and mechanisms that were affected in grapevine infected with GRBV and in modulating different host responses in leaves and berries. This research serves as a foundation for a better understanding of the molecular interactions in this plant-geminivirus pathosystem.

**Supplementary Information:**

The online version contains supplementary material available at 10.1186/s12864-025-11539-4.

## Background

Small RNAs (sRNAs) are the central component of all RNA silencing pathways in plants due to their regulatory roles in a multitude of developmental and physiological processes, as well as in response to biotic and abiotic stresses [1–5]. Among the different classes of sRNAs characterized in virus-infected plants, microRNAs (miRNAs) and small-interfering RNAs (siRNAs) are important contributors to both transcriptional (TGS) and post-transcriptional gene silencing (PTGS). In addition, plants have evolved sRNA-mediated silencing, or RNA interference (RNAi) as a natural defense strategy to counter viral infections [6, 7]. RNAi is an evolutionarily conserved antiviral mechanism mediated by virus-derived siRNAs (vsiRNAs) [8, 9]. Multiple studies have demonstrated that viral infections in plants are closely tied to the accumulation of vsiRNAs, which are consequently able to directly target and silence viral RNA [10–12].

Unlike siRNAs, miRNAs originate solely from an organism’s nuclear genome, either in dedicated *MIR* genes, or within introns of other specific genes. Following transcription, miRNA precursors form a stem loop structure and are processed by a Dicer or Dicer-like protein (DCL) into a miRNA duplex. This duplex is then exported from the nucleus, after which it associates with an argonaut protein forming the miRNA-induced silencing complex (miRISC). The miRNA guides the miRISC to a complementary segment of a target plant mRNA. The miRISC then initiates PTGS and the resulting degradation of the target mRNA [13]. Sequencing of the plant degradome has been highly effective at identifying the resulting fragments of this degradation and can be used to validate miRNA targets within plant transcriptomes [14–17].

Extensive research into and characterization of miRNAs, including target identification, have revealed that miRNAs are not only important regulators in plant processes ranging from development to environmental stress responses [18, 19], but that miRNA expression itself can vary significantly in response to different conditions, including viral disease [20, 21].

Grapevine red blotch disease (GRBD), caused by Grapevine red blotch virus (GRBV, genus *Grablovirus* and family *Geminiviridae*) [22–26] is an economically important viral disease affecting wine grapes (*Vitis vinifera* L.) in different grapevine-growing regions ( [27], and cited references). The disease affects both the yield and quality of grapes produced by infected vines leading to significant reduction in income to growers [28–30]. Virus-infected vines produce contrasting symptoms in red- and white-fruited *V. vinifera* cultivars. In red-fruited cultivars, initial symptoms on leaves consist of small, irregular, red-colored areas between major veins that expand and coalesce as the season advances to become reddish or reddish-purple irregular blotches. In addition, some red-fruited cultivars show red-colored primary, secondary, and tertiary veins. Interestingly, despite GRBV infecting grapevines systemically and being detectable throughout the season, visual symptoms only begin to appear after véraison, which represents the onset of ripening [31]. Following véraison, disease symptoms begin to appear in infected vines primarily on mature leaves at the basal portion of the shoots. As the season advances, symptoms can appear progressively in maturing leaves positioned farther up on shoots [32]. In contrast, white-fruited cultivars may show mild symptoms that are less conspicuous with irregular chlorotic areas between major veins, sometimes accompanied by necrosis around the leaf margins.

**Fig. 1:**
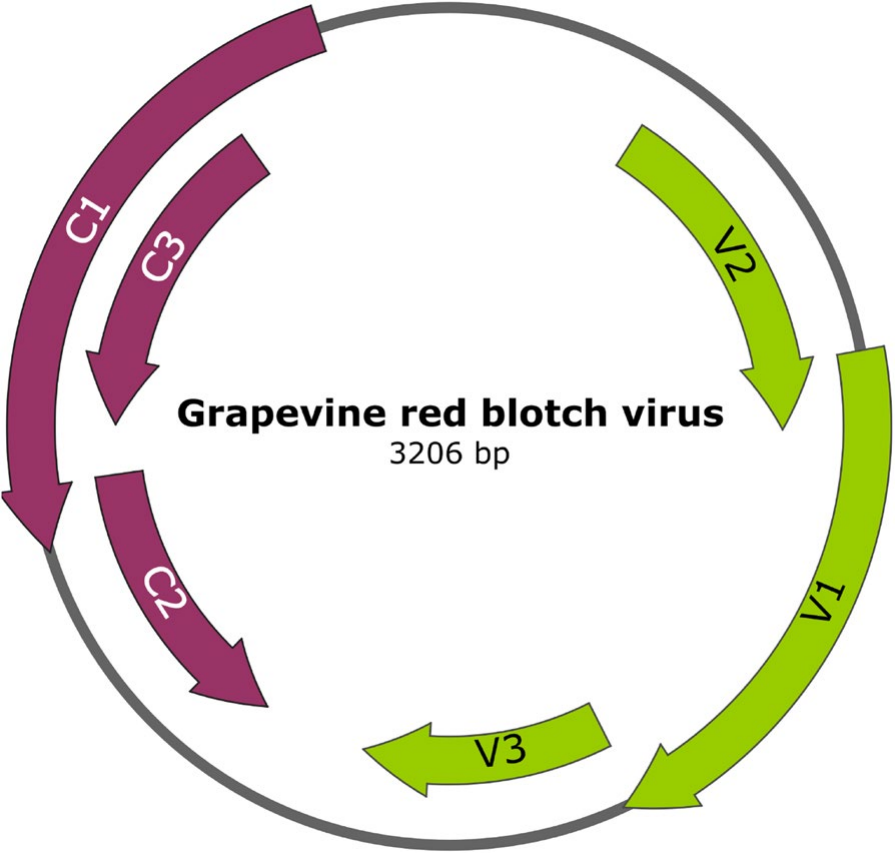
Grapevine red blotch virus (GRBV) Genome. Locations of viral (green) and complementary (red) sense ORFs in GRBV genome. Created with SnapGene software [37] and the GRBV RefSeq annotation [22]

GRBV has a circular single-stranded DNA genome of around 3,200 nucleotides and encodes six clearly defined open reading frames (ORFs), with three overlapping ORFs (C1, C2, and C3) in the complementary sense and the other three (V1, V2, and V3) in the viral sense (Fig. 1) [33, 34]. ORF V1 encodes the predicted viral coat protein (CP), although CP was not detected in infected plants nor virions observed via electron microscopy. The V2 and V3 ORFs are suggested to encode movement proteins based on similarities with other monopartite geminiviruses [35]. The C1 ORF was predicted to encode RepA, and a spliced transcript encoded by C1 and C2 was predicted to encode Rep. The function of the C3 ORF, which is located internal to and within the same frame as the C1 ORF, is yet to be determined. Recently, the proteins encoded by the C2 and V2 ORFs were identified as suppressors of post-transcriptional gene silencing [36].

In recent years, many studies utilizing sRNA profiling have been conducted in virus-infected plants to identify both plant- and virus-derived sRNAs and gather information on associated plant-viral interactions [38–41]. These studies were useful not only for building up an information base that can be used to interpret and contextualize various molecular interactions between plants and their viral pathogens, but also for discovering potential tools for managing viral plant diseases and to further elucidate the molecular biology and population genetics of viruses [9].

Many of these studies, however, were focused on annual plants in a defined set of controlled environments. In recent years, these types of studies were extended to perennial crops such as grapevine [38, 42–45]. Unlike in annual crops, the dynamics of sRNAs in virus-infected perennial crops that stay on the ground for many years are influenced by seasonal and environmental changes. Though recent studies have advanced knowledge on the epidemiology and impacts of GRBV [27, 46–48], little is known regarding virus-host interactions at the molecular level. Because red blotch disease symptoms are produced in a phenological stage-specific manner during each season, it can be hypothesized that the dynamics of virus-host interactions are distinct between asymptomatic pre- and symptomatic post-véraison stages. Among many molecular interactions, investigating the regulatory roles of sRNAs with regard to both infection status and phenological stage could lead to deeper insights into the molecular pathways contributing to symptom development in field-grown grapevines.

In this study, high-throughput sRNA sequencing was used to analyze the sRNAome in two different tissue types across two phenological stages from GRBV-infected grapevines (*Vitis vinifera* cv. Merlot) from a commercial vineyard. Profiling both plant miRNAs and viral siRNAs, along with their targets, provided a foundation for understanding the role of sRNA-dependent regulatory mechanisms in GRBV-grapevine interactions.

## Materials and Methods

### Plant material

Leaf and berry samples were collected from a commercial vineyard (45°52′07″N, 119°46′30″W) planted in 2008 with own-rooted *Vitis vinifera* cv. Merlot (clone 15) vines and maintained by the grower using standard viticultural practices. Based on grower feedback, the vineyard block was planted with compromised planting stock resulting in the introduction of GRBV into the block at the time of planting. Grapevines were selected for this study in pairs with each pair consisting of one vine showing GRBD symptoms and an adjacent, asymptomatic vine in the same row. They were selected such that each pair of grapevines is from a different row within the vineyard block. Candidate vines were tested initially for GRBV via PCR assays following the methodology described in Adiputra et al. 2018 [32], to ensure that the symptomatic vines were positive for GRBV and that the asymptomatic vines were negative.

Three pairs of vines, with each pair consisting of one symptomatic, GRBV-positive vine and one asymptomatic, GRBV-negative vine, were selected for this study. Both leaf and berry samples were collected separately from individual vines at pre-véraison (early July 2015) and post-véraison (September 2015). This resulted in eight distinct sampling categories based on the combination of timepoint, tissue type, and infection status. These sampling categories have been denoted according to the abbreviations, P (pre-véraison), PO (post-véraison), L (leaves), B (berries), H (GRBV-negative), and D (GRBV-positive), such that “PLH” would reference pre-véraison leaf samples from GRBV-negative vines.

The samples were snap-frozen in liquid N_2_ immediately after harvesting and transported in liquid N_2_ to maintain the integrity of the samples until they were stored at -80 °C. Individual leaf and berry samples collected from each vine were considered as one biological replicate for downstream applications.

### Preparation of sRNA Libraries, Sequencing, and Mapping of sRNAs

Small RNAs were extracted from frozen leaf and berry tissues using a mirPremier® microRNA isolation kit (Sigma-Aldrich, MO) by following the protocol provided by the manufacturer. Separate sRNA libraries were generated from these samples following the protocol discussed in Alabi et al. [42] and Li et al. [49]. The quantity of sRNA preparations was assessed based on 260 nm/280 nm OD values using a NanoDrop 2000c spectrophotometer and RNA integrity was measured using the 2100 Bioanalyzer system (Agilent Technologies, SantaClara, CA). Preparations with 260/280 absorbance ratio from 1.8–2.0 and an RNA integrity number (RIN) higher than 7.0 were used for library preparations. High quality sRNA samples were shipped to BGI Genomics [50] for library construction and Illumina 50SE sequencing using the Hiseq 4000 system (RRID:SCR_016386). Read quality was checked with FastQC. *V. vinifera* miRNAs were identified and mapped to the 12X.v2 grapevine reference genome assembly [51], alongside several other databases including premiRBase21, Rfam, Silva, TIGR Plant Repeat Databases, and Repbase, using BOWTIE version 1.0.0 and following the methods and parameters described in Suo et al. [52]. Following mapping, all miRNAs without a minimum of 10 counts in at least one sample were removed. The remaining miRNAs were normalized to reads per million mapped reads (RPM). However, one replicate (PLD2) was excluded from further analysis due to poor quality.

sRNA reads were also mapped to virus sequences under 23 kb in length available in GenBank [53]. Two different methods for mapping the sRNA reads to the database were tested. In the first method, reads were first mapped to the Ensembl grapevine genome assembly (version 12X.v2), and then the unmapped sequences were mapped to the viral database. In the second method, the trimmed sequences were mapped directly to the viral database. Since there was minimal difference between the two methods, the second method was used for all subsequent analyses. Mapping was performed in CLC Genomic Workbench version 21.0.5 requiring perfect matches and excluding reads which mapped non-specifically. The sRNA reads were also exclusively mapped to the grapevine genome with the same method for the determination of the length profile of host-specific sRNAs.

### sRNA length distribution analysis

The abundances of different lengths of sRNAs which mapped to the *Vitis vinifera* and GRBV genomes were determined using a custom python script (see availability of data and materials) to parse tables of mapped reads exported from CLC Genomics Workbench for each sample, count the number of reads of each length from 18–28 nucleotides, and assemble these counts into a table. R Statistical Coding Language version 3.6.3 [54] and the “dplyr”, “car”, “xtable”, and “gridExtra” packages were used for the statistical analysis. Read counts were normalized by sample via the ‘scale’ function. The ‘aov’ function was used to perform two-way ANOVAs looking at the effects of both infection and véraison status on the prevalence of each read length. Levene tests were performed using the ‘leveneTest’ function. Tukey post-hoc analyses were performed on all of the ANOVAs using the ‘TukeyHSD’ function. Results were organized and exported using the ‘xtable’, ‘rbind’, and ‘grid.table’ functions.

### Grapevine degradome sequencing for target identification

The protocols described by Li et al. [15, 55] were used to prepare and sequence four degradome libraries, representing GRBV-positive and GRBV-negative leaf samples taken at both pre- and post-véraison. Trimmomatic version 0.39 [56] was used to prepare the four libraries for analysis. The first six nucleotides of each read were cropped. Reads shorter than 23 nucleotides were excluded and reads over 28 nucleotides in length were trimmed down to 25 nucleotides. Additionally, an overrepresented sequence (> 25%) which mapped to *V. vinifera* chloroplastic DNA was removed prior to analysis.

CleaveLand version 4.5 [57] was used to identify targets in the degradome libraries. The CleaveLand script was modified with regard to the section involving Bowtie, such that the default parameters (-k 1 -best) were altered (-a -m 12 -best -strata) to allow for reads to map to multiple loci, so long as they did not map to more than twelve separate loci.

The cDNA annotation was obtained from Ensembl Plants version 54 [58]. The TAS3 sequences LOC100244732 and LOC104879803 were added manually from NCBI. Grape miRNA sequences were obtained from miRBase [59]. The vvi-tas3 sequences were derived from LOC100244732 and LOC104879803 based on their homology to known 21nt tas3 sequences. Additionally, a series of custom Python version 3.8.3 [60] scripts was used to parse the sRNAs which mapped to the GRBV genome, record the fifty most abundant unique sequences from each GRBV-positive sample, and combine them into a non-redundant list, which was then used in the CleaveLand pipeline. This was chosen as an alternative to running all of the unique mapped sequences due to time and computational constraints. In total, 83 unique sRNA sequences were included. Custom Python version 3.8.3 [60] scripts were used to determine statistics such as the 9nt, 10nt, and 11nt cleavage counts, the degradome peak ranks, valid reads, the total number of degradome reads per transcript, and percent reads valid.

Due to the lack of gene descriptions on the *V. vinifera* annotation used, BLASTp (from BLAST + version 2.10.1) was used to match proteins from *V. vinifera* with those from *A. thaliana* with an e-value cutoff of 1e-10. Protein sequences for both species were obtained from Ensembl Plants version 54. Each *V. vinifera* gene was assigned a homologue from *A. thaliana* based on the strongest BLAST hit (if any). Annotations for the *A. thaliana* genes were derived from the Ensembl Plants version 54 annotations. For genes without *A. thaliana* homologues, target gene functions were determined through use of NCBI’s conserved domain search function.

### miRNA differential expression analysis

The differential expression analysis was performed in the R Statistical Coding Language version 3.6.3 [54] using the “edgeR” (version 3.28.1) package by Bioconductor [61], with a generalized likelihood ratio model. Prior to the analysis, miRNAs that did not have more than one read in at least six samples were removed. Instead of the normalized read counts (RPM), the raw read counts were used in conjunction with the ‘calcnormfactors’ function in edgeR. The ‘DGEList’, ‘glmFit’, and ‘glmLRT’ functions were used to conduct the differential expression analysis, which was performed as a series of pairwise comparisons between groups utilizing the ‘makecontrasts’ function. To determine significance, a p-value cutoff of < 0.05 and log(FC) cutoff of >|1.0| was used.

### Mapping sRNAs to GRBV Genome

CLC Genomics Workbench was used to map the sRNA reads which had been previously mapped to the GRBV genome in the viral database to a gene-annotated version of the GRBV genome. This was done, as opposed to utilizing the entirety of the trimmed reads, to prevent the inclusion of the previously excluded non-specific reads. The results of this mapping were analyzed using the “edgeR” package by Bioconductor [61] in R. Reads were normalized using the ‘calcnormfactors’ function. Reads mapping to the GRBV ORFs were analyzed for differential expression using the ‘DGEList’, ‘glmFit’, amd ‘glmLRT’ functions. This was done via a series of pairwise comparisons between experimental groups set up using the ‘makecontrasts’ function. To determine significance, an FDR cutoff of < 0.05 and log(FC) cutoff of >|0.2| was used.

## Results

### PCR assays of grapevine samples

The petioles of mature leaves were collected from Merlot vines exhibiting GRBD symptoms as well as adjacent, asymptomatic vines and tested for an array of common grapevine-infecting viruses, including GRBV, by PCR and RT-PCR. The diagnostic results were used to select three symptomatic vines that tested positive only for GRBV and three asymptomatic vines, tested negative for viruses, adjacent to GRBV-positive vines, for a total of six vines. High-throughput sequence (HTS) analysis of small RNAs described below confirmed the presence of GRBV only in symptomatic vines. No other viruses were detected in these samples by HTS analysis, confirming the diagnostic results mentioned above. In addition, two viroid species (Hop stunt viroid and Grapevine yellow speckle viroid 1) were found in both GRBV-positive and negative vines (Table S3). Further analysis of these viroids was not pursued in this study.

### sRNA sequencing and mapping

A total of 24 sRNA libraries were constructed and sequenced using the Illumina sequencing platform. These 24 libraries represented three biological replicates for leaf and berry samples harvested at pre- and post-véraison stages from symptomatic, GRBV-positive vines and asymptomatic, GRBV-negative vines. The clean and mapped read numbers in each library are shown in Table S1. Per each of the eight sampling categories, there was an average of roughly 598,000 unique reads (Table S2).

**Fig. 2:**
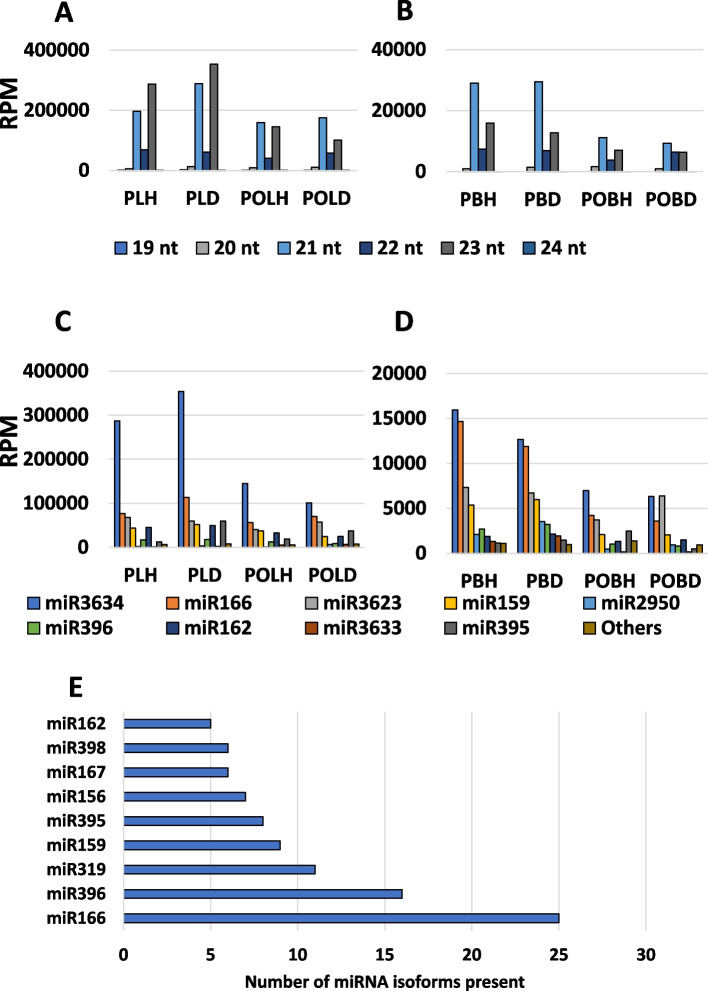
Abundances of miRNAs by length, family, and number of isoforms. Length distribution of miRNAs identified from the (**A**) leaves and (**B**) berries from GRBV-positive and negative vines collected during pre- and post-véraison. Reads were normalized by RPM. In leaf samples, 23nt reads were the most abundant during pre-véraison, but 21nt reads were the most abundant during post-véraison. In berries, 21nt reads were the most abundant in samples collected during pre- and post-véraison. Family-wise distribution of miRNAs in (**C**) leaves and (**D**) berries from GRBV-negative and GRBV-positive samples collected during pre- and post-véraison. Reads were normalized by RPM. **E** Number of distinct miRNA isoforms detected from miRNA families. Reads from the miR3634 family were the most abundant in every sampling category except for berry samples at post-véraison from GRBV-positive vines, where miR3623 reads were more abundant. The overabundance of miR3634 family reads was due to the isoform miRNA3634a-3p. This overabundance also contributed to the high 23nt read abundance in leaves. Sampling categories are denoted according to the following abbreviations: pre-véraison [P], post-véraison [PO], leaves [L], berries [B], GRBV-negative [H], GRBV-positive [D]

Across all libraries, there were a total of 560,479,564 raw reads. Following the removal of low-quality reads and trimming for adapter sequences, a total of 23,129,077 clean reads remained. These reads were mapped to the 12X.v2 grapevine reference genome assembly [51], alongside several other databases (Table S2), as described in Suo et al. [52]. Following the removal of reads shorter than 19 and longer than 24 nucleotides, 2,210,559 reads remained that were successfully mapped to miRNA sequences. After filtering out reads with less than 10 counts present in a single sample, the remaining reads were mapped to 140 unique miRNAs belonging to 42 miRNA families using the methodology described in Suo et al. [52]. Among the mapped reads, 21 and 23 nt-long reads were the most common (Fig. 2A and B). Following normalization (RPM), eight miRNAs had over 1,000 RPM across all replicates (Fig. 2C and D). One specific isoform, miR3634a-3p, had a disproportionately high abundance relative to the other miRNAs identified. The isoform miR3634a-3p had 45,335 RPM between all replicates, while the next most prevalent isoform, miR3623a-3p, had 10,406 RPM (Figure S1). Three miRNA families (miR166, miR319, and miR396) were represented by more than ten distinct isoforms. Six other miRNA families (miR156, miR159, miR162, miR167, miR395, and miR398) were represented by five or more isoforms (Fig. 2E).

The trimmed sRNA reads from all 24 libraries were also mapped to a GenBank virus database containing all plant viruses using CLC Genomics Workbench version 21.0.5. Mapping parameters were set to exclude any nonspecific results and disallow mismatches. The results are summarized in Table S3.

**Fig. 3:**
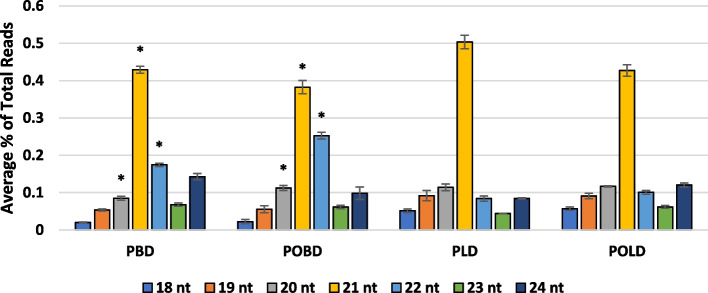
Virus-derived siRNA abundance by length. Length distribution of sRNA reads mapping to GRBV genome with no mismatches and no nonspecific reads plotted as the percentage of total reads mapped. Comparisons were made in R between sampling categories for each sRNA. Asterisks show significant differences (*p* < 0.05) between sample categories based on véraison. Significant differences based on tissue type are not shown. sRNA reads ranging from 25–28 nt long are not shown due to low abundance. Sampling categories are denoted according to the following abbreviations: pre-véraison [P], post-véraison [PO], leaves [L], berries [B]

For reads mapped to the GRBV genome, 25nt and longer reads made up minimal (< 2%) portions of the overall read distribution. The 21nt reads were the most common in all sampling categories. Notably, 21nt sRNAs were significantly less abundant in post-véraison berries than in pre-véraison berries (Fig. 3). The 20nt and 22nt reads showed an inverse relationship, where they were more abundant in post-véraison berry samples. There was also a relatively low abundance of 24nt vsiRNAs compared to 21nt and 22nt vsiRNAs.

Substantial quantities of reads from sRNA libraries from GRBV-positive vines mapped to the GRBV genome with high coverage, while reads from GRBV-negative samples had very few reads mapped to GRBV. No other viruses had substantial read abundance and high coverage in libraries from any samples.

### Small RNA Target identification using Degradome Analysis

Four degradome libraries were prepared and sequenced from GRBV-positive and GRBV-negative leaf samples from both pre- and post-véraison. Across all four libraries, there were 66,492,906 raw reads. The first six nucleotides were trimmed from all raw reads with Trimmomatic 0.39 [56]. Then the reads with less than 23nt were removed, and reads longer than 28nt were trimmed to 25 nt. An overrepresented sequence mapping to chloroplastic DNA was also removed, resulting in a total of 38,204,856 clean reads, which were used to identify miRNA targets using CleaveLand version 4.5 [57]. This was performed using the Ensembl Plants version 54 [58] annotation of the *Vitis vinifera* transcriptome. In the pooled degradome from leaves of GRBV-negative vines, a total of 9,241,719 reads were reported to have at least one alignment to the transcriptome. In the degradome from leaves of GRBV-positive vines, this total was 14,059,040 reads. Between both pools, only 75,081 reads were omitted due to the use of the (-m) argument.

Target gene annotations were determined by assigning homologues from the Ensembl Plants version 54 *A. thaliana* annotation. This allowed for the identification of a total of 58 conserved targets for 18 miRNA families (Table 1). Additionally, three targets for tasiRNA3 were identified. T-plots for select targets are shown in Figure S3. The remaining targets were screened according to strict criteria of having greater than 10 total valid reads and greater than 5% valid reads. Using this approach, 41 novel targets for 19 miRNA families were identified, over half of which exhibited greater than 25% valid reads in one or both pooled degradomes (Table 2, Table S4).

**Table 1:** Degradome validation of miRNA family targets and target functions

Family	Transcript	Site	Allen	GRBV- Leaves			GRBV + leaves			Target function
				Valid	%	Cat	Valid	%	Cat	
miR156	Vitvi10g04328_t001	885	2	112	14.58	2	167	10.9	2	squamosa_promoter_binding_protein-like_3
	Vitvi12g00280_t001	704	0	1	0.82	4	NA	NA	NA	squamosa_promoter_binding_protein-like_4
	Vitvi11g00909_t001	1116	0.5	1	6.67	4	1	7.69	4	squamosa_promoter_binding_protein-like_2
	Vitvi17g00473_t001	1242	0.5	1	25	4	NA	NA	NA	Squamosa_promoter-binding_protein
	Vitvi01g01837_t001	1330	0.5	3	25	1	1	5.56	4	Squamosa_promoter-binding_protein
	Vitvi01g01660_t001	1354	0.5	1	6.25	4	2	10.53	4	squamosa_promoter_binding_protein-like_2
	Vitvi01g01678_t001	891	0.5	NA	NA	NA	1	100	4	Squamosa_promoter-binding_protein
	Vitvi17g00100_t001	1223	0.5	NA	NA	NA	1	33.33	4	Squamosa_promoter-binding_protein
miR159	Vitvi13g01266_t001	1732	2.5	13	48.15	0	13	44.83	0	myb_domain_protein_33
miR160	Vitvi18g00337_t001	1304	0.5	2	1.36	2	4	1.71	2	auxin_response_factor_17
	Vitvi13g02058_t001	1337	1	3	4.05	2	2	1.67	2	auxin_response_factor_16
	Vitvi06g00272_t001	1719	1	1	3.57	4	NA	NA	NA	auxin_response_factor_10
	Vitvi08g01033_t001	2190	0.5	NA	NA	NA	1	0.71	4	auxin_response_factor_16
miR162	Vitvi15g00864_t001	3261	2	3	0.45	2	1	0.1	4	dicer-like_1
miR164	Vitvi17g00622_t001	997	1	3	21.43	0	4	12.9	0	NAC_domain_containing_protein_100
	Vitvi19g01484_t001	809	2	3	6.52	2	3	3.61	2	NAC_domain_containing_protein_1
miR166	Vitvi09g00310_t001	562	1	7	3.45	0	23	7.28	0	Homeobox-leucine_zipper_family_protein
	Vitvi06g00276_t001	574	1.5	16	6.5	2	28	6.97	2	Homeobox-leucine_zipper_family_protein
	Vitvi13g00609_t001	763	1.5	15	23.81	0	11	13.58	0	Homeobox-leucine_zipper_family_protein
	Vitvi10g00913_t001	806	1.5	19	9.79	0	36	12.46	0	Homeobox-leucine_zipper_family_protein
	Vitvi04g00287_t001	1036	1.5	7	11.11	0	23	19.49	0	Homeobox-leucine_zipper_family_protein
miR167	Vitvi04g00824_t001	1841	4	165	35.56	2	226	34.35	2	auxin_response_factor_8
	Vitvi10g00854_t001	3613	4	8	4.88	0	14	6.48	0	auxin_response_factor_8
	Vitvi12g00102_t001	3855	4	14	20	0	21	22.58	0	auxin_response_factor_6
miR168	Vitvi17g01218_t001	702	4	129	11.92	0	190	13.59	0	argonaute 1
miR169	Vitvi08g01883_t001	1510	3	1	2.5	4	2	3.45	2	nuclear_factor_Y,_subunit_A1
	Vitvi09g00133_t001	1373	1.5	2	1.4	3	NA	NA	NA	nuclear_factor_Y,_subunit_A3
	Vitvi08g00292_t001	1357	3	9	2.24	2	21	3.4	2	nuclear_factor_Y,_subunit_A10
miR171	Vitvi04g01247_t001	599	0	61	15.1	0	70	14.4	2	GRAS_family_transcription_factor
	Vitvi15g00680_t001	1601	0	53	4.22	0	83	4.32	0	GRAS_family_transcription_factor
	Vitvi02g00536_t001	1664	1	128	10.48	0	114	6.75	0	GRAS_family_transcription_factor
miR172	Vitvi13g00529_t001	1348	1	18	17.14	0	17	9.24	0	related_to_AP2.7
	Vitvi06g00360_t001	1825	1.5	3	9.38	1	1	3.13	4	related_to_AP2.7
	Vitvi07g01706_t001	1934	1	22	8.12	0	26	4.96	0	APETALA2
miR319	Vitvi06g01139_t001	1254	4	20	41.67	0	36	48.65	0	myb_domain_protein_33
	Vitvi12g00219_t001	786	4.5	1	0.13	4	4	0.38	3	TCP_family_transcription_factor_4
miR393	Vitvi00g04585_t001	1516	1	2	18.18	1	4	26.67	0	F-box/RNI-like_superfamily_protein
	Vitvi07g00248_t001	1637	1	29	37.18	0	33	31.73	0	F-box/RNI-like_superfamily_protein
	Vitvi14g04156_t001	2014	1	2	13.33	1	4	20	0	F-box/RNI-like_superfamily_protein
	Vitvi14g01482_t001	2172	1	98	7.52	0	116	5.54	0	auxin_signaling_F-box_3
miR395	Vitvi18g00363_t001	77	1.5	NA	NA	NA	4	1.47	2	sulfate_transporter_2;1
miR396	Vitvi02g00796_t001	250	2.5	6	1.32	2	7	1.03	2	leucine_zipper_transcription_factor_16
	Vitvi08g01498_t001	548	3	4	100	3	7	58.33	0	growth-regulating_factor_4
	Vitvi02g00239_t001	671	3	3	13.64	1	3	17.65	1	growth-regulating_factor_8
miR398	Vitvi14g02607_t005	69	4	367	21.79	0	625	22.84	0	copper/zinc_superoxide_dismutase_1
	Vitvi06g01349_t001	477	6.5	4	1.57	2	5	1.32	2	copper/zinc_superoxide_dismutase_2
	Vitvi02g00444_t001	773	7	8	2.4	2	14	2.71	2	copper_chaperone_for_SOD1
	Vitvi11g01445_t001	77	4	18	9.78	2	11	5.76	2	blue-copper-binding_protein
miR828	Vitvi17g00822_t001	360	4	2	33.33	1	4	80	0	myb_domain_protein_66
	Vitvi02g01732_t001	478	1	23	88.46	0	23	88.46	0	myb_domain_protein_66
	Vitvi14g03020_t001	492	4	3	75	0	7	100	3	myb_domain_protein_23
miR858	Vitvi14g00974_t001	347	5.5	4	2.58	2	2	0.57	4	myb_domain_protein_4
	Vitvi11g00097_t001	362	2.5	22	25.88	0	50	36.5	0	myb_domain_protein
	Vitvi06g00414_t001	369	4.5	1	4.55	4	NA	NA	NA	myb_domain_protein_59
	Vitvi07g00393_t001	433	5	14	10.85	0	13	6.57	0	myb_domain_protein_12
	Vitvi09g00112_t001	451	4.5	41	28.47	0	66	27.16	0	myb_domain_protein_7
	Vitvi04g00160_t001	303	4.5	NA	NA	NA	3	75	0	myb_domain_protein_66
	Vitvi08g01797_t001	324	5	NA	NA	NA	3	0.55	3	myb_domain_protein_5
tasiRNA3	Vitvi01g01759_t001	1203	2	4	0.48	2	5	0.42	2	auxin_response_factor_2
	Vitvi10g00510_t001	1431	0	3	0.08	3	7	0.13	3	auxin-responsive_factor
		1638	0	4	0.11	3	11	0.2	2	auxin-responsive_factor
	Vitvi17g00036_t001	1738	0.5	17	2.79	2	15	1.62	2	auxin_response_factor_2

Cleavage position (site) and Allen score (Allen) for miRNA targets. Number of valid reads, percentage of total reads that are valid, and category value for degradome validation in both GRBV-negative (GRBV-) and GRBV-positive (GRBV +) leaves. Target functions based on *A. thaliana* annotation and further supported by NCBI BLAST results

**Table 2:** Target function annotations for novel targets

Query	Transcript	Arabidopsis homologue	Gene description
miR159	Vitvi12g00209_t001	AT2G42570.1	TRICHOME_BIREFRINGENCE-LIKE_39
miR167	Vitvi03g00206_t001	AT3G12500.1	basic_chitinase
miR168	Vitvi09g02081_t001	ATMG00510.1	*Complex 1 49 kDa superfamily; respiratory-chain NADH dehydrogenase, 49 Kd subunit (cl21493)
			Interval 16-1155; E 2.01e-62; Bit 205
	Vitvi09g02090_t001	ATMG00510.1	*Complex 1 49kDa superfamily; respiratory-chain NADH dehydrogenase, 49 Kd subunit (cl21493)
			Interval 16–1155; E 2.01e-62; Bit 205
miR3476	Vitvi03g00706_t001	AT1G04945.2	HIT-type_Zinc_finger_family_protein
	Vitvi13g01803_t001	AT1G04945.2	HIT-type_Zinc_finger_family_protein
	Vitvi15g04391_t001	AT1G04945.4	HIT-type_Zinc_finger_family_protein
	Vitvi15g04396_t001	AT1G04945.4	HIT-type_Zinc_finger_family_protein
	Vitvi15g04403_t001	AT1G04945.4	HIT-type_Zinc_finger_family_protein
	Vitvi16g00437_t001	AT3G06530.2	ARM_repeat_superfamily_protein
miR3623	Vitvi18g00591_t001	AT5G49650.1	xylulose_kinase-2
	Vitvi16g00880_t001	AT2G45590.1	Protein_kinase_superfamily_protein
miR3624	Vitvi10g00241_t001	AT4G16380.3	Heavy_metal_transport/detoxification_superfamily_protein
	Vitvi10g00245_t002	AT4G16380.2	Heavy_metal_transport/detoxification_superfamily_protein
	Vitvi08g01651_t001	AT3G57000.1	nucleolar_essential_protein-like_protein
	Vitvi13g01167_t001	AT3G57000.1	nucleolar_essential_protein-like_protein
miR3629	Vitvi13g01758_t001	AT5G56710.1	Ribosomal_protein_L31e_family_protein
miR3633	Vitvi03g00203_t001	AT4G35250.1	NAD(P)-binding_Rossmann-fold_superfamily_protein
	Vitvi03g04485_t001	NA	*Protein of Unknown Function
	Vitvi10g04333_t001	NA	*Protein of Unknown Function
	Vitvi07g01217_t001	AT4G02600.2	Seven_transmembrane_MLO_family_protein
miR3634	Vitvi19g04623_t001	NA	*Ank 2 superfamily; Ankyrin repeats (3 copies); cl39094;
			Interval 212–370; E 3.27e-04; Bit 38.95
miR3635	Vitvi03g01290_t001	AT3G12750.1	zinc_transporter_1
miR395	Vitvi06g01295_t001	AT2G28000.1	chaperonin-60alpha
miR396	Vitvi01g00876_t001	AT1G59640.1	transcription_factor_BIG_PETAL_P_(BPE)
	Vitvi06g04411_t001	AT2G27970.1	CDK-subunit_2
	Vitvi06g01133_t001	AT1G50420.1	scarecrow-like_3
	Vitvi02g01038_t001	AT5G26742.3	DEAD_box_RNA_helicase_(RH3)
miR397	Vitvi09g00019_t001	AT1G72330.1	alanine_aminotransferase_2
miR477	Vitvi07g02021_t001	AT1G04290.1	Thioesterase_superfamily_protein
	Vitvi17g00218_t001	AT1G18210.2	Calcium-binding_EF-hand_family_protein
	Vitvi11g00481_t001	AT5G20200.1	nucleoporin-like_protein
miR482	Vitvi18g02996_t001	NA	*Protein of Unknown Function
	Vitvi09g01354_t001	AT1G51580.1	RNA-binding_KH_domain-containing_protein
	Vitvi13g04689_t001	AT3G14470.1	NB-ARC_domain-containing_disease_resistance_protein
	Vitvi14g01187_t001	AT5G16120.4	alpha/beta-Hydrolases_superfamily_protein
miR5139	Vitvi18g01220_t001	AT3G14430.1	GRIP/coiled-coil_protein
miR828	Vitvi14g04439_t001	NA	*Protein of Unknown Function
miR894	Vitvi14g04415_t001	AT3G18280.1	Bifunctional_inhibitor/lipid-transfer_protein/
			seed_storage_2S_albumin_superfamily_protein
miR894	Vitvi14g04418_t001	AT3G18280.2	Bifunctional_inhibitor/lipid-transfer_protein/
			seed_storage_2S_albumin_superfamily_protein

Target functions are based on *A. thaliana* annotation and further supported by NCBI BLAST results. Target functions marked with an asterisk (*) either lacked an annotated function in the *A. thaliana* annotation of the *A. thaliana* homologue or lacked an *A. thaliana* homologue altogether. In this case, functions were annotated using the NCBI Conserved Domain Search function. In some cases, no matching domains were found, in which case they were marked as proteins of unknown function

Using the methods described above, putative targets were also identified for several sRNAs mapped to the GRBV genome (Table 3). Valid targets were manually curated based on relaxed cutoffs (> 2% Valid Reads and > 4 10nt reads). The t-plots and transcript alignments of the selected targets were then considered before finalizing the list of valid targets (Figure S3). These analyses were performed twice, once using the degradome data from healthy samples, and once using the degradome data from infected samples (Table S5). This was done to determine if cleavage events from GRBV-specific sRNAs also had support in GRBV-negative samples, which could indicate that the observed degradome support may not be tied with the vsiRNA-guided cleavage event. Degradome support for the identified targets did vary based on which degradome data was used. The overwhelming majority of potential targets either lacked substantial alignment at the 5’ end or had poor degradome support. In the end, only fourteen targets for eight different vsiRNAs were identified.

**Table 3:** GRBV-derived siRNAs with putative targets in *V. vinifera* transcriptome

sRNA	Sequence	Start-Stop	ORF
grbvasRNA1	AAACGACGTGTCTGGTGGAGG	1246–1266	V1
grbvasRNA2	ACGACTGGGAGGAGTTCTGCC	761–781	V2
grbvasRNA3	AGGTGTTGTGCTTCCGTCGGA	946–966	V1
grbvasRNA4	ATGATGGGTTAGGGGATGAGG	389–409	V2
grbvasRNA5-3p	ATGGGCTATATCATTGGGAAT	2222–2202	C2
grbvasRNA6-3p	ATGTGGCAATGACTCCTGCGG	1192–1172	V1
grbvasRNA7a	TTGTGGTGATGATGATGGGTT	378–398	V2
grbvasRNA7b	TTTGTGGTGATGATGATGGGT	377–397	V2

Sequences of identified grbvasRNAs with potential targets in the *V. vinifera* genome and locations within the GRBV genome. The “-3p” suffix indicates that the detected vsiRNA sequence was the reverse complement of the associated sequence in the GRBV reference genome. The other identified vsiRNAs aligned normally in the 5’-3’ direction

For ease of reference, the eight identified vsiRNAs were assigned identifiers grbvasRNA1-7. Instead of using grbvasRNA8, grbvasRNA7a and grbvasRNA7b were used, since the two sequences differed only by a single nucleotide (Table 3). Interestingly, all but one of these eight vsiRNAs are mapped to ORFs V1 and V2, while only grbvasRNA5 is located within the complementary-sense C1 ORF. It is worth noting that, among the eight identified vsiRNAs, targets were identified for the 3’-5’ forms of grbvasRNA5 and grbvasRNA6. This was expected for grbvasRNA5, as it is within the C2 ORF, which is normally transcribed in the 3’-5’ direction. However, grbvasRNA6 is derived from the V1 ORF, which is transcribed in the 5’-3’ direction. This indicates that for grbvasRNA6 to accumulate, it would need to have a source other than the normal transcription of the V1 ORF, such as read-through transcription in the 3’-5’ direction.

For the selected vsiRNA targets, gene annotations were identified using NCBI’s Conserved Domain Search function [62]. These targets included proteins involved in intracellular transport, photosynthesis, apoptosis, transcription, translation, DNA/RNA recognition, binding, and maintenance (Table 4, Table S6).

**Table 4:** Identified targets of GRBV-derived siRNAs

Query	Target	Target Function (Conserved Domain Search)
grbvasRNA1	Vitvi01g00128_t001	Mu homology domain (MHD) of adaptor protein (AP) coat protein I (COPI) delta subunit, cd09254; delta subunit of the F-COPI complex, N-terminal domain, cd14830
	Vitvi01g00128_t002	C-terminal domain of adaptor protein (AP) complexes medium mu subunits and its homologs, cl10970, member cd09254: AP_delta-COPI_MHD; delta subunit of the F-COPI complex, N-terminal domain, cd14830
grbvasRNA2	Vitvi06g00398_t001	Ribosomal L29 protein, pfam00831
	Vitvi07g01275_t001	photosystem II oxygen-evolving enhancer protein 1, PLN00037
grbvasRNA3	Vitvi19g00148_t001	Apoptosis inhibitory protein 5 (API5), pfam05918
grbvasRNA4	Vitvi09g00707_t001	RNA recognition motif (RRM) superfamily, cl17169, member cd12288: RNA recognition motif (RRM) found in plant proteins related to the La autoantigen; inosine-5'-monophosphate dehydrogenase, cl33447, member PLN02274, inosine-5'-monophosphate dehydrogenase; inosine-5'-monophosphate dehydrogenase cl36546, member PTZ00314, inosine-5'-monophosphate dehydrogenase, Provisional
grbvasRNA5	Vitvi15g01427_t001	Zn-finger in Ran binding protein and others, pfam00641
	Vitvi15g01427_t002	Zn-finger in Ran binding protein and others, pfam00641
grbvasRNA6	Vitvi19g01704_t001	Superfamily II DNA and RNA helicase [Replication, recombination and repair], COG0513
grbvasRNA7a	Vitvi17g00483_t001	PPR repeat family, pfam13041; maturation of RBCL 1, cl33664 member PLN03218; Uncharacterized protein cl23818 member PRK00976: methanogenesis marker 12 protein
	Vitvi18g01006_t001	Chloroplast import apparatus Tic20-like, cl15935 member TIGR00994: 3a0901s05TIC20 chloroplast protein import component, Tic20 family
	Vitvi18g01006_t002	Chloroplast import apparatus Tic20-like, cl15935 member TIGR00994: 3a0901s05TIC20 chloroplast protein import component, Tic20 family
	Vitvi18g01006_t003	Chloroplast import apparatus Tic20-like, cl15935 member TIGR00994: 3a0901s05TIC20 chloroplast protein import component, Tic20 family
grbvasRNA7b	Vitvi11g00285_t001	DNA-binding domain in plant proteins such as APETALA2 and EREBPs, smart00380

### Expression of miRNAs

To determine miRNA expression levels between GRBV-infected and noninfected samples and between the two phenological stages, read counts were examined using the package ‘edgeR’ by Bioconductor in R Statistical Language [54]. Due to a large number of miRNAs that were detected in very low counts and only in certain categories or replicates, miRNAs lacking at least one count in a minimum of six different samples were excluded from the analysis, leaving 99 unique miRNAs. Significant differential expression was determined through use of the Likelihood Ratio Model. After applying cutoffs (*P*-Value < 0.05; |log2(FC)|> 1.0), a total of 63 unique miRNAs belonging to 28 different families were significantly differentially expressed between the comparisons made. *P*-value was chosen as the cutoff metric for this analysis, instead of the more conservative FDR value, due to the extremely high variance in the data (common dispersion > 0.8). In the following sections, the term “differentially expressed” is used to indicate miRNAs that passed these cutoffs regarding a specific comparison (i.e., GRBV + vs. GRBV- or pre- vs. post-véraison).

### Differences in miRNA Expression Between GRBV-negative and GRBV-positive Samples

Upon comparison between GRBV-positive and GRBV-negative samples, a total of 41 miRNAs belonging to 18 different miRNA families showed differential expression (Table S7). Three miRNAs, miR166ax, miR3624a-3p, and miR482aw, were differentially expressed in both pre- and post-véraison berries. Similarly, miR396j was differentially expressed in both pre- and post-véraison leaves. Including miR396j, twelve miRNAs were only differentially expressed in the leaves, while 24 were only differentially expressed in berries. Pre-véraison berries and post-véraison leaves each had differentially expressed miRNAs belonging to a single family that was uniquely represented within the corresponding category. These families were miR156 and miR2950, in berry and leaf samples, respectively. Three more families, miR159, miR162, and miR166, were similarly uniquely represented by differentially expressed isoforms in post-véraison berries. The miRNA3624 family was differentially expressed within both pre- and post-véraison berry samples, but not in leaf samples. Among the miRNAs that were significantly differentially expressed, 28 miRNAs belonging to fifteen families were upregulated and fifteen miRNAs from eight different families were downregulated in response to viral infection (Table S7). There was no overlap between leaf and berry sample categories in down regulated miRNAs. No downregulated miRNAs were observed in leaf samples from the pre-véraison stage. Pre-véraison berries had two down-regulated miRNAs, miR166be and miR6478a, while post-véraison berries and post-véraison leaves had seven downregulated miRNAs each. Isoforms of miR156, miR395, and miR3624 were only down-regulated in post-véraison berries. The miR396 family had down-regulated members in all post-véraison leaf and berry samples. The miR166 family had members that were down-regulated in all samples except in pre-véraison leaves. Additionally, isoforms of miR159 and miR319 were only downregulated in post-véraison leaves.

**Fig. 4:**
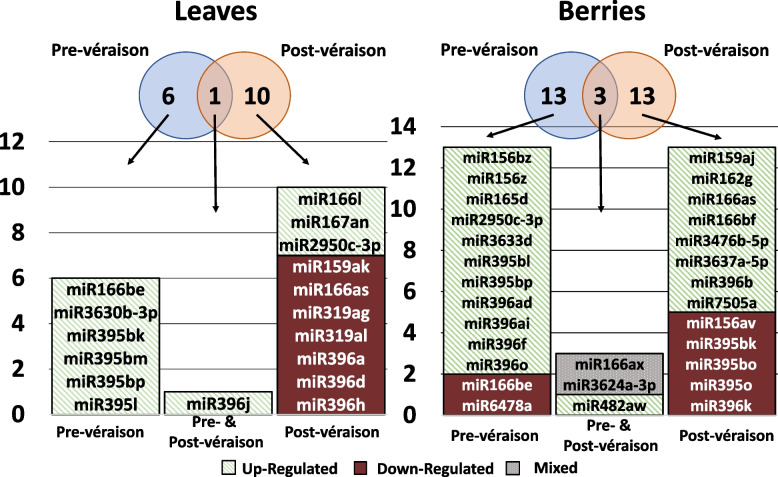
Differential expression of miRNAs in GRBV-negative vs. GRBV-positive vines during pre- and post-véraison stages. The Venn diagram shows the number of miRNA isoforms differentially expressed in response to infection by GRBV in leaves and berries, either in pre-véraison, post-véraison, or at both stages. Bar charts show the number, direction, and the identity of differentially expressed miRNAs in response to infection, either in pre-véraison, post-véraison, or at both stages. Red indicates miRNAs that were downregulated in GRBV-positive relative to GRBV-negative samples, and green indicates miRNAs that were up-regulated in GRBV-positive relative to GRBV-negative samples, either in pre-véraison, post-véraison, or at both stages. Grey indicates that miR166ax and miR3624a-3p were up-regulated during pre-véraison but down-regulated during post-véraison

Upregulation of several miRNAs occurred in both pre- and post-véraison samples (Fig. 4). In pre-véraison berries, eleven miRNAs were upregulated in infected relative to healthy samples. This includes miR3624a-3p, which was down-regulated in post-véraison berries. Eight miRNAs were differentially expressed in post-véraison berries only. The miR482aw was upregulated in both pre-and post-véraison berries while miR396j was upregulated in both pre- and post-véraison leaves. Aside from miR482aw and miR396j, pre-véraison leaves showed six upregulated miRNAs, belonging to miR166, miR395, and miR3630. Post-véraison leaves showed upregulation of three miRNAs, namely miR166l, miR167an, and miR2950-3c. Members of miR166 and miR396 were upregulated in all four categories of samples in GRBV-positive vines relative to GRBV-negative vines. Members of miR395 were upregulated in all pre-véraison samples and members of miR3630 and miR167 families were only upregulated in leaf samples taken at pre- and post-véraison, respectively. Additionally, members of miR156, miR165, miR3624, and miR3633 were exclusively upregulated in pre-véraison berry samples, while members of miR159, miR162, miR3476, miR3637, and miR7505 were uniquely upregulated in post-véraison berries (Fig. 4). In contrast, miR166ax and miR3624a-3p were up-regulated during pre-véraison and down-regulated during post-véraison. The fold change, *p*-values, and FDR values for differential expression of miRNAs are shown in Table S7.

### Phenological stage-specific differences in miRNA expression in leaves and berries

**Fig. 5:**
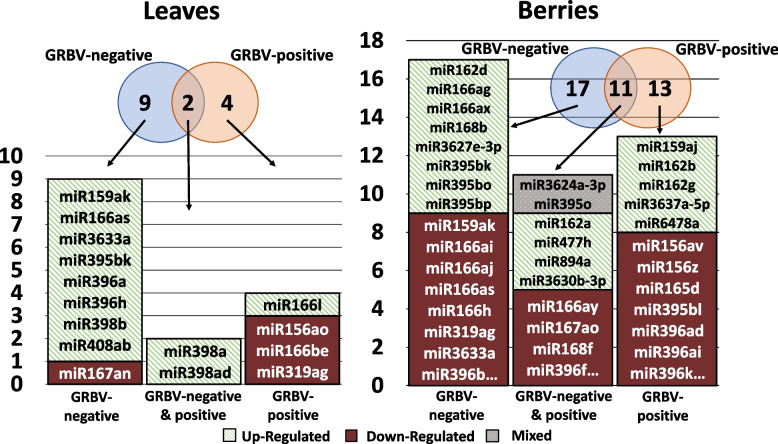
Differential expression of miRNAs during pre-véraison vs. post-véraison in leaf and berry samples. The Venn diagram shows the number of miRNAs differentially expressed in post-véraison relative to pre-véraison in leaves and berries of GRBV-negative vines, GRBV-positive vines, or both GRBV-negative and positive vines. The bar chart shows miRNAs differentially expressed in post-véraison relative to pre-véraison in GRBV-negative vines, GRBV-positive vines, or both GRBV-negative and positive vines. Red indicates miRNAs that were downregulated post- relative to pre-véraison, and green indicates miRNAs that were up-regulated post- relative to pre-véraison, either in GRBV-positive or GRBV-negative vines, or in both. Grey indicates that miR395o and miR3624a-3p were upregulated in GRBV-negative berries and downregulated in GRBV-positive berries

Upon comparison of miRNA expression patterns in leaf and berry samples from pre-véraison relative to post-véraison, there were 50 miRNAs belonging to 20 different families which showed differential expression (Table S8). Among them, nine miRNAs belonging to six different families showed differential expression only in the leaves. Among these six families, miR398 and miR408 did not have any isoforms that showed differential expression in berry samples. Six miRNAs belonging to six different families were differentially expressed in both leaves and berries, while the remaining 35 miRNAs were only differentially expressed in the berries. Additionally, fifteen miRNAs belonging to miR156, miR159, miR162, miR165, miR166, miRR395, miR396, miR3637, and miR6478 families were differentially expressed only in GRBV-positive samples. Eighteen miRNAs belonging to miR159, miR162, miR166, miR167, miR168, miR3627, miR3633, miR395, miR396, and miR398 families were differentially expressed only in GRBV-negative samples (Fig. 5).

### Differences in Abundance of vsiRNAs Specific to GRBV ORFs

To determine if the GRBV-derived sRNAs were particularly associated with specific regions of the GRBV genome (Fig. 1), sRNA reads were mapped to the NCBI gene-annotated version of the GRBV genome (accession NC_022002.1). To gauge whether tissue type and phenological stage had an impact on the relative abundance of sRNAs mapped to each ORF encoded by the virus, they were analyzed using the differential expression pipeline described above. Out of 1,360,633 reads that mapped to the GRBV genome, 1,302,547 sRNA reads (> 95%) mapped to known ORFs. The number of reads that mapped to individual ORFs in each sample are shown in Table S5. The highest number of vsiRNAs mapped to the V3 ORF, which had almost double the normalized reads (RPM) when compared to the other ORFs. The high abundance of V3-specific vsiRNAs was consistent in leaves and berries from both pre- and post-véraison stages.

**Fig. 6:**
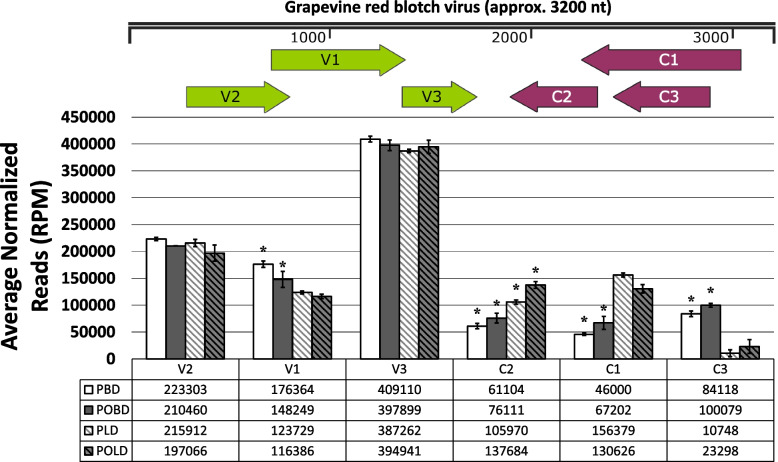
Abundance of vsiRNAs specific to GRBV ORFs. A linearized depiction of the circular GRBV genome (5’ to 3’) showing ORF locations within the GRBV genome. Green, right-facing arrows indicate ORFs transcribed in the 5’-3’, viral sense. Red, left-facing arrows indicate ORFs transcribed in the 3’-5’, complementary sense. This depiction was created using SnapGene software [37] and the RefSeq GRBV annotation [22]. Abundance (RPM) of sRNAs which mapped to specific GRBV ORFs. Asterisks above individual bars indicate significant differences between pre- and post-véraison within a tissue type. In berries, sRNAs mapping to V1 were significantly less abundant and sRNAs mapping to C1-3 were significantly more abundant. In leaves, sRNAs mapping to C2 were significantly more abundant in post- relative to pre- véraison. Sampling categories are denoted according to the following abbreviations: pre-véraison [P], post-véraison [PO], leaves [L], berries [B], GRBV-positive [D]

Four different ORFs exhibited differential sRNA abundance between pre- and post-véraison berry samples, while only a single ORF exhibited differential expression between pre-and post-véraison leaf samples. In berries, sRNAs that mapped to C1, C2, and C3 ORFs were significantly more abundant in post-véraison relative to pre-véraison, while the sRNAs specific to the V1 ORF were significantly less abundant in post-véraison relative to pre-véraison. In leaves, sRNAs specific to the C2 ORF were significantly more abundant in post-véraison relative to pre-véraison samples (Fig. 6).

## Discussion

GRBV is a monopartite geminivirus and is economically important among the four DNA viruses currently reported infecting grapevines [63]. Among the GRBV-derived siRNAs, 21nt reads were the most common in all sampling categories, which is consistent with previous observations from other viral pathosystems, including grapevine leafroll-associated virus 3 [42]. This observation suggests that DCL4 could be the primary dicer involved in the generation of GRBV-derived vsiRNAs, since DCL4 is responsible for the generation of 21nt sRNAs [9, 64]. Interestingly, 20nt and 22nt vsiRNAs were significantly more abundant during post-véraison compared to pre-véraison stages. This could be indicative of a shift in DCL2 activity across the growing season, as DCL2 is the primary dicer involved in generating both 20nt and 22nt sRNAs [9, 64, 65]. sRNA-mediated silencing driven by long sRNAs (~ 24nt) is likely to act at the DNA methylation level, whereas smaller sRNAs (20-22nt) act via RNA degradation [1]. Indeed, the production of 24nt siRNAs by DCL3 has been directly tied to the RNA-directed DNA methylation (RdDM) pathway [66, 67]. The greater abundance of 21nt and 22nt vsiRNAs, relative to the low abundant 24nt vsiRNAs, may suggest that most antiviral silencing is targeting GRBV transcripts and that the action of the RdDM pathway is unlikely to play a major role in silencing GRBV. This contrasts with observations made in geminivirus-infected *Arabidopsis*, which have demonstrated the importance of RdDM in antiviral defense [68, 69]. Our findings may suggest that the RdDM pathway is not the primary defense strategy against DNA viruses in plants, as has been previously suggested [9].

vsiRNAs have been shown to target host mRNA transcripts, leading to symptom expression ( [70], and cited references). In this study, we identified eight distinct vsiRNAs with a total of 14 targets in the grapevine transcriptome. Of them, five possess functional domains directly tied to chloroplast function. An additional target, containing a pentatricopeptide repeat (PPR), is also connected to chloroplast function, as PPR proteins are known to be involved in the posttranscriptional regulation (RNA maturation, editing, intron splicing) of chloroplastic and mitochondrial genes [71]. Additionally, GRBV has been demonstrated to lower photosynthetic rates in infected vines [27]. In other viral systems, including Rice stripe virus and Southern rice black-streaked dwarf virus, vsiRNAs targeting host genes encoding chloroplastic proteins have been implicated as a mechanism leading to the development of chlorotic symptoms [72, 73]. It is possible that grbvasRNA2 and grbvasRNA7a are, in part, playing a role in chloroplast function leading to decreased photosynthesis in GRBV-infected grapevines. Since this study used a limited number of leaf and berry samples at pre- and post-véraison from a single cultivar (cv. Merlot), future studies are needed with multiple cultivars for a better understanding of GRBV-grapevine interactions and to elucidate phenotypic differences in disease symptoms across different wine grape cultivars.

Among the many miRNAs reported in this study, miR3634a-3p was highly abundant, in pre- and post-véraison leaf and berry samples from both GRBV-positive and negative vines (Figure S4). However, miR3634a-3p was relatively more abundant in pre-véraison leaf samples from both GRBV-positive and negative vines (Fig. 2C). The high abundance of miR3634a-3p has biological significance, since this miRNA targets the grapevine transcript Vitvi19g04623_t001 (Table 2), which contains 3 copies of an ankyrin repeat domain from the Ank 2 superfamily. Ankyrin repeat domains are involved in a wide array of different protein functions, including transport, cell–cell signaling, and various regulatory processes [74]. Previously, an isoform of miR3634 in grapevine was found to target a transcript for an E3 ubiquitin ligase [75]. Identification of an additional target of a completely unrelated function suggests that the miR3634 family may regulate a variety of functions in grapevine.

The miR159, miR166, miR395, miR396, and miR3623 families were also highly expressed in leaf and berry samples across pre- and post-véraison stages (Fig. 2), like in previous grapevine miRNA studies [42, 75, 76]. The miR166 family targets a class III Homeodomain leucine-zipper protein (Table 1), which could play a role in wood formation [42, 77]. Though previous studies have shown miR166 displaying upregulation in response to viral infection [21, 42], we have observed variable responses of miR166 family members upon infection with GRBV. In particular, miR166ax displayed distinct profiles during pre- and post-véraison, suggesting phenological stage-specific responses to GRBV infection (Table S7). Ten members of the miR396 family showed variable responses to GRBV in pre- and post-véraison leaf and berry samples (Table 2.3a).

The miR396 family regulates the growth regulating factors (GRFs), which are involved in many developmental processes, ranging from general cell proliferation to flower, fruit, and seed formation [76, 78–80]. Our analysis has validated two GRFs, specifically GRF4 and GRF8 as targets for miR396 (Table 1). These two GRFs are primarily involved in cell proliferation of leaf tissue [80]. The upregulation of miR396j in GRBV-infected leaf samples from pre- and post-véraison (Table 2.3a) suggest that miR396j could regulate two GRF factors thereby affecting leaf growth in GRBV-infected plants, which, in turn, could contribute to overall reduction in plant vigor [27]. We also identified novel targets for the miR396 family, including the transcription factor Big Petal P (BPEp), Cyclin-Dependent Kinase (CDK) subunit 2, and Scarecrow-like protein 3 (Table 2), all of which are involved in one or more aspects of plant development or growth [81–83]. Scarecrow-like protein 3, specifically, promotes gibberellin signaling, which in turn induces an array of different plant growth and development processes [83, 84]. The up-regulation of different miR396-family miRNAs in both pre- and post-véraison berries in response to GRBV infection warrants further investigation, as this upregulation could be modulating the gibberellin signaling pathway, contributing to the reduction in berry development.

The miR156 family is also known to regulate proteins involved in plant development, specifically the SQUAMOSA promoter-binding protein-like (SPL) transcription factors, which have been shown to play roles in both fruit ripening and stress response [85, 86]. In this study, miRNAs belonging to the miR156 family exhibited up-regulation in response to GRBV infection in berries during pre-véraison, but down-regulation in post-véraison (Table S7). These observations would indicate that the miR156 family may contribute to impeded berry development in GRBV-infected vines.

The miR167 family is known to target auxin response factors 6 and 8 (ARF6 and ARF8) (Table 1) [87]. ARF6 is a positive regulator of photosynthetic processes, sugar accumulation, and fruit ripening [88], and both ARFs are positive regulators of jasmonic acid biosynthesis [89]. Increased expression of miR167 has been shown to lead to defective flower development in tomato [87]. In this study, several members of the miR167 family were differentially expressed in response to GRBV infection, with miR167an being upregulated during post-véraison (Table S7). Thus, miR167an could play a role in the negative impacts on photosynthetic activity, fruit ripening, and jasmonic acid synthesis as previously reported in GRBV-infected vines [27, 90].

Based on degradome data, a protein homologous to the *A. thaliana* Xylulose kinase-2 protein, which is integral to the isoprenoid biosynthesis pathway [91], was identified as a target of miR3623 (Table 2). The miR3623 family has also been linked to the regulation of disease resistance genes [92]. Our findings show that miR3623-family miRNAs were not differentially expressed in response to GRBV-infection, which could suggest that, in this plant-pathogen system, the expression of miR3623 is tied more closely to the regulation of isoprenoid biosynthesis and that the regulation of disease resistance proteins may be a secondary function.

Members of the miR482 family are also involved in the regulation of disease resistance R genes [92–94]. It has been suggested that the miR482 family could reduce the fitness costs associated with inefficient or non-functioning R genes, protecting against their misexpression, and allowing more freedom for genetic variation [95]. In this study, miR482aw was upregulated only in berries of infected vines in both pre- and post-véraison stages (Table S7). This regulation may play a role in suppressing the expression of host resistance genes against GRBV infection in a tissue type-specific manner, but this warrants further investigation.

Increased expression of miR395 in vines infected with grapevine leafroll-associated virus 3 has been reported [38]. The miR395 family is known to regulate transcripts of ATP sulfurylase and sulfate transporter 2;1 in plants. The lowered expression of these transcripts leads to the accumulation of sulfate in plant tissue [96, 97]. Sulfate and sulfate-derived compounds are important contributors to stress tolerance in plants and play a role in plant defense strategies against pathogens [38, 98]. miR395 members were also up-regulated in response to GRBV infection during pre-véraison in both leaf and berry tissue which in turn could play a role in modulating sulfate accumulation and metabolism in GRBV-infected vines.

Two closely related miRNA families, miR159 and miR319, are known to target the MYB33 and MYB65 gene families (Table 1) [99, 100] that are associated with several aspects of plant growth and development [101, 102] and stress tolerance [103]. In this study, miR159ak, miR319ag, and miR319al showed down-regulation in response to GRBV infection in post-véraison leaves. This is in contrast to previous studies that observed higher expression of miR159 and miR319 in response to viral infection in other plant systems [21, 104, 105].

Additionally, some MYBs, such as VvMYB114, have been shown to regulate anthocyanin accumulation in grapevines [106]. VvMYB114 is known to be regulated by miR828 and miR858. These miRNAs also target MYB4, MYB5, MYB7, MYB12, MYB23, MYB59, and MYB66 family proteins (Table 1). However, the lack of differential expression of miR828 and miR858 in pre- or post-véraison leaf samples would suggest that these miRNAs are not involved in the modulation of MYBs involved in anthocyanin biosynthesis.

## Conclusions

In this study, we have identified 41 miRNAs that were differentially expressed in response to GRBV infection (Table S7). In addition, 50 miRNAs showed differential expression between pre- and post-véraison (Table S8). We also found 58 targets for conserved miRNAs (Table 1), as well as 40 novel targets for grapevine miRNAs (Table 2), all supported by degradome analysis. These reported differentially expressed miRNAs in *V. vinifera* cv. Merlot vines infected with GRBV offers important insights into our understanding of grapevine-GRBV interactions under field conditions. Overall, the miRNA and vsiRNA analysis in this study lay a foundation for future research into the mechanisms of the interactions between *Vitis vinifera* and GRBV.

One limitation to our approach is that this study only profiled miRNAs and vsiRNAs using sequencing-based approaches and lacks independent validations of differentially expressed vsiRNAs and miRNAs as well as their targets. However, we have used three replicates from three independent pairs of both infected and non-infected vines for miRNA and vsiRNA analysis. This offers reasonable confidence in our overall analysis. Further research involving a larger set of samples across phenological stages from different wine grape cultivars infected with GRBV would strengthen these reported results.

## Supplementary Information

Supplementary Material 1.

Supplementary Material 2.

## Data Availability

The raw sRNA and degradome sequencing data are available under the BioProject accession PRJNA1129059, or at the following link: https://www.ncbi.nlm.nih.gov/sra/PRJNA1129059. The associated SRA accession numbers for the sequencing files are SRR29616259-86. The python script which was used to parse read length data is available at [https://github.com/noah-ault/Ault-GRBV-sRNA-Dynamics.git].
