# Supplementary material for: Dynamics of small RNAs in a red-fruited wine grape cultivar infected with Grapevine red blotch virus
Source: BMC Genomics. 2025 Apr 29;26:417. doi: 10.1186/s12864-025-11539-4 (PMC12038946; doi:10.1186/s12864-025-11539-4)
Supplement: Supplementary file 2 — Supplementary Material 2. [file 12864_2025_11539_MOESM2_ESM.docx]

**Figure S1: miRNA abundance by isoform**

Abundance of individual miRNAs detected across small RNA libraries normalized to RPM. Only includes miRNAs with over 100 total RPM across all libraries. Reads for miR3634-3p made up most mapped reads. Reads mapping to miR159, miR162, miR166, miR395, miR3623 families were also highly abundant relative to other families.

**Figure S2: miRNA target prediction T-plots**

**A**


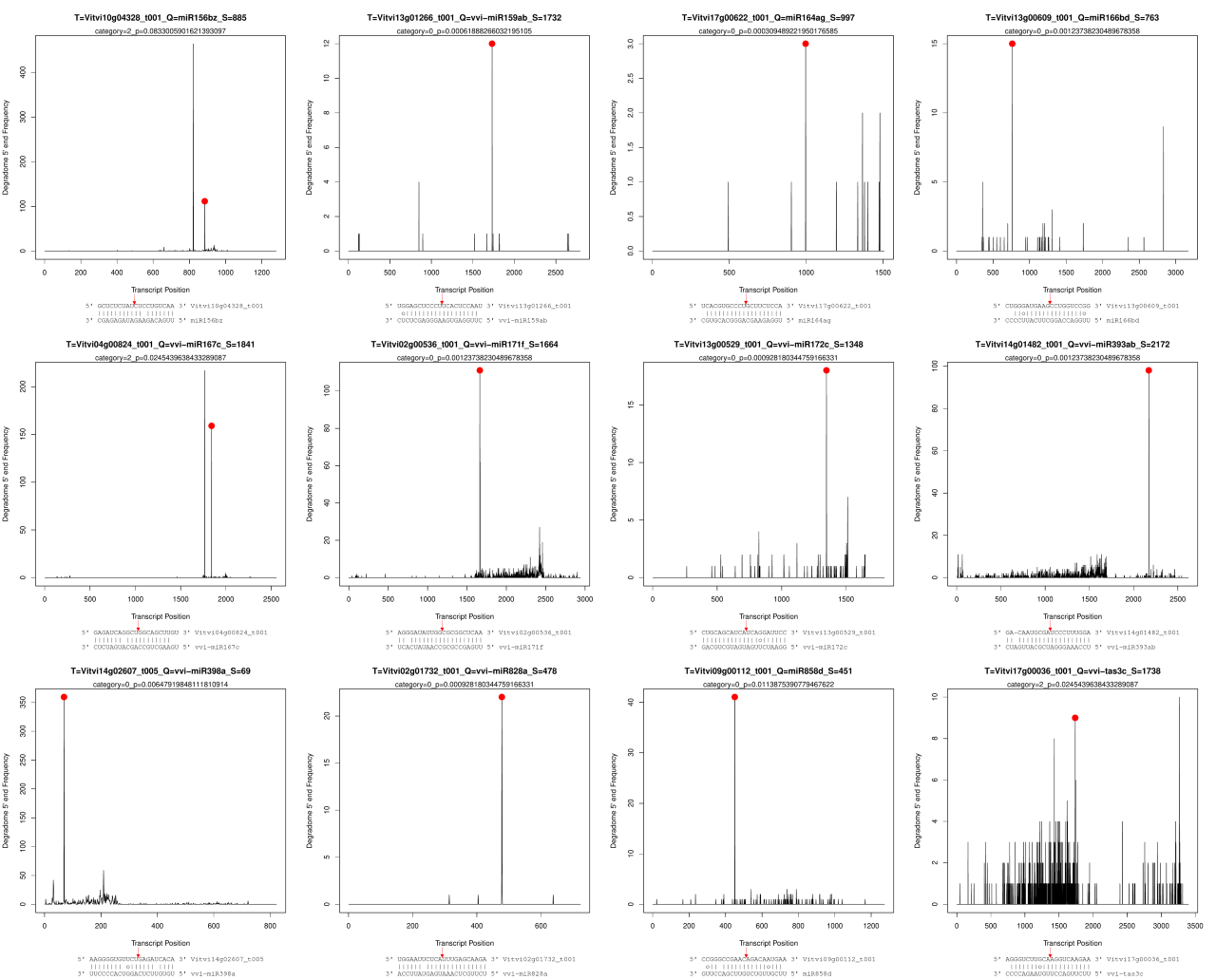

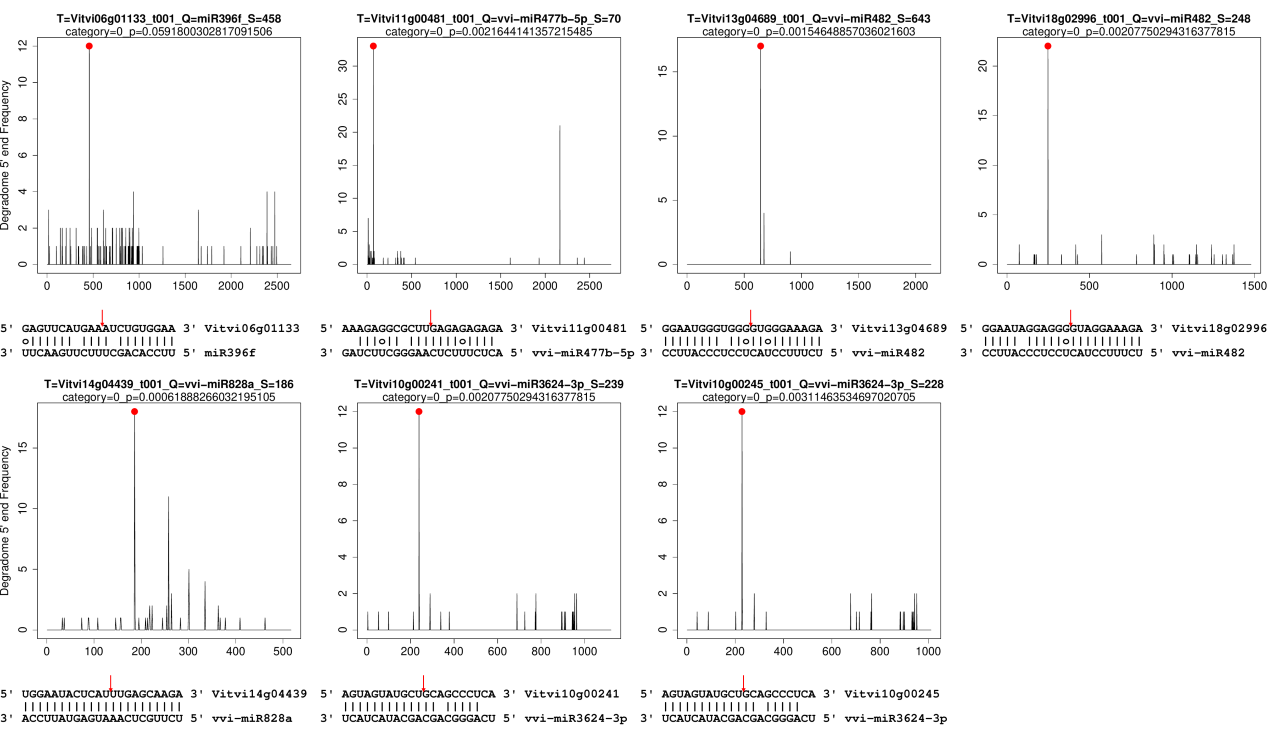


**B**

T-plots, transcript alignments, and cleavage site locations for select, conserved (A) and novel (B) sRNA targets in the grapevine transcriptome. The red dots indicate the abundance of 5’ends lining up in the correct location within the predicted transcript. Other peaks indicate other locations 5’-ends from the degradome data mapped to in the transcript. The red arrows indicate the predicted cleavage location in the alignment of the transcript and the miRNA.

**Figure S3: GRBV-derived sRNA target prediction T-plots**


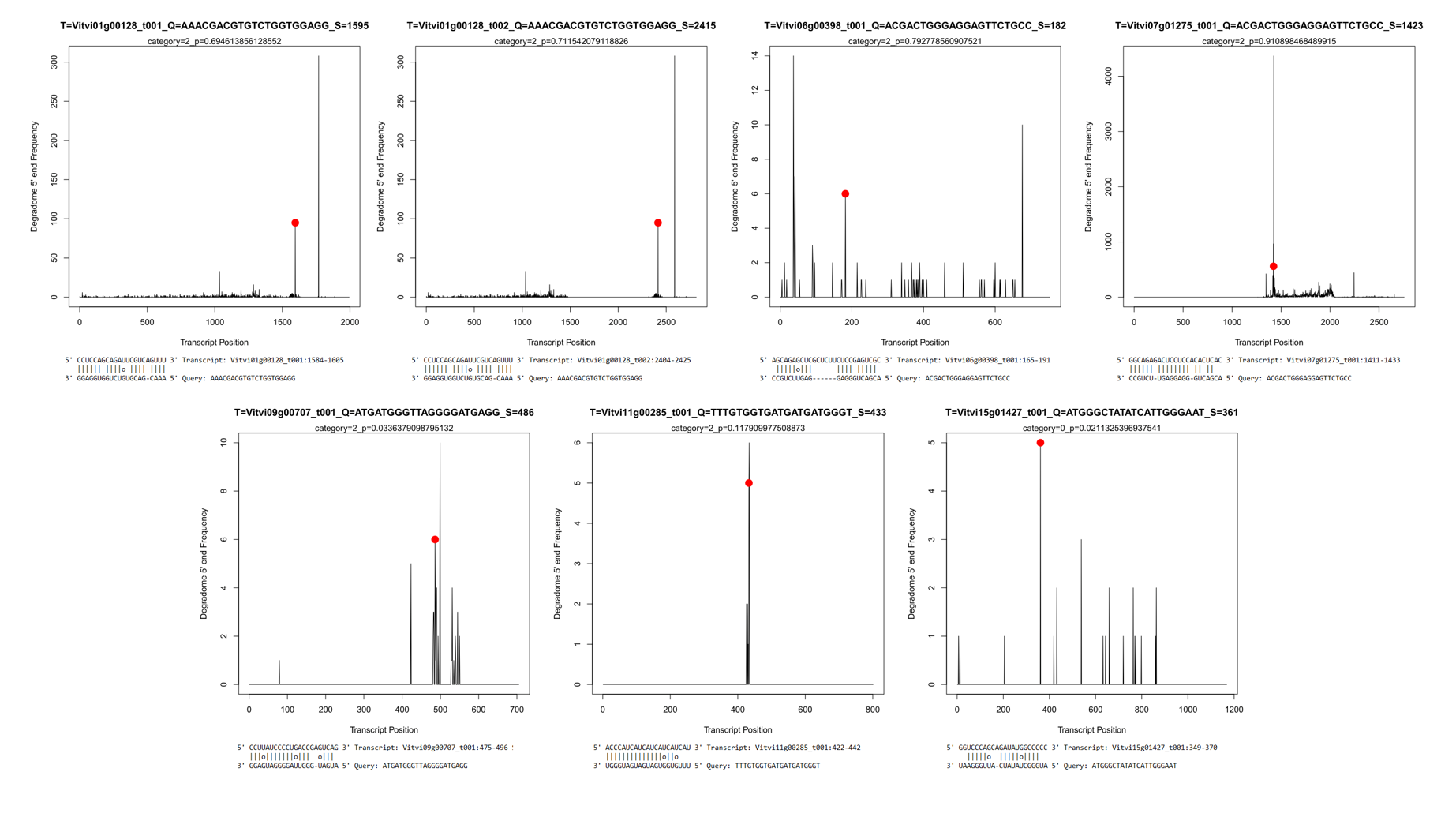

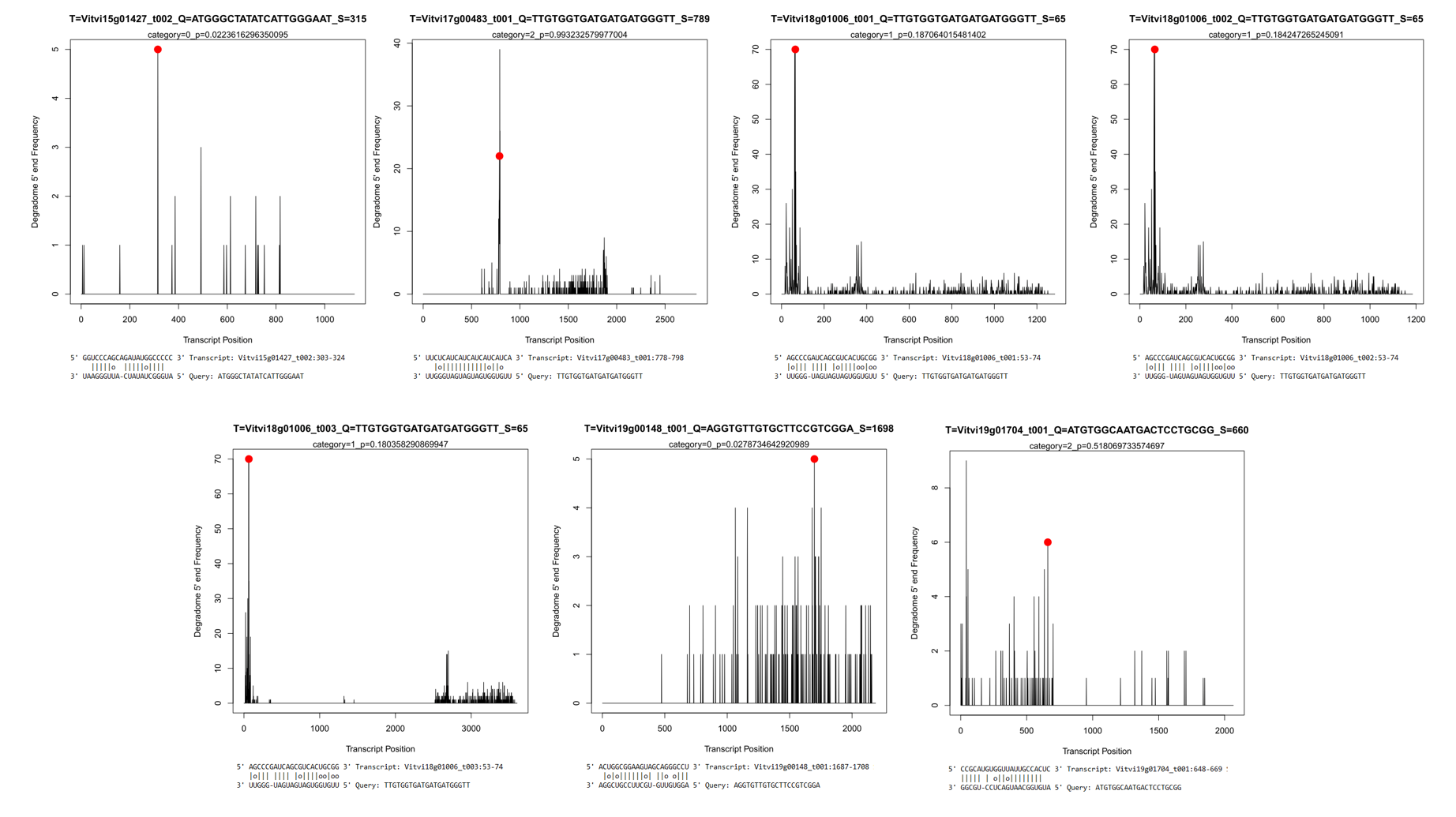


T-plots and transcript alignments for grbvasRNA targets in the grapevine transcriptome. Individual peaks represent the occurrence of degradome reads with 5’ ends aligning to a specific location within the grapevine transcript. Red dots are placed at the tip of the peak located at the predicted 10nt slice site.

**
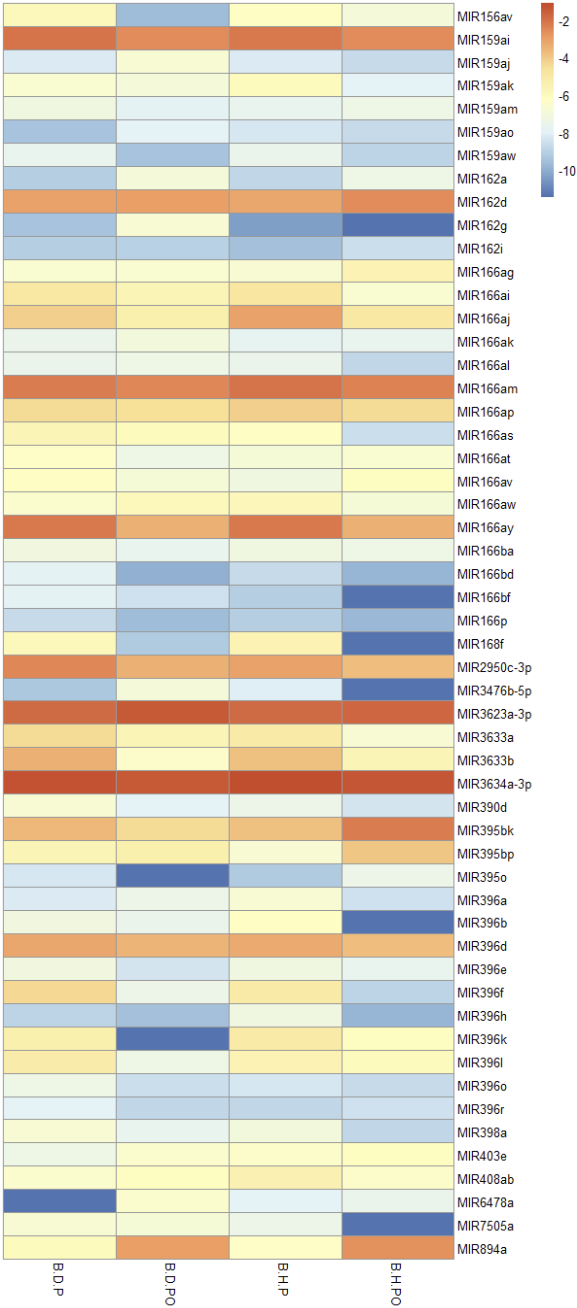

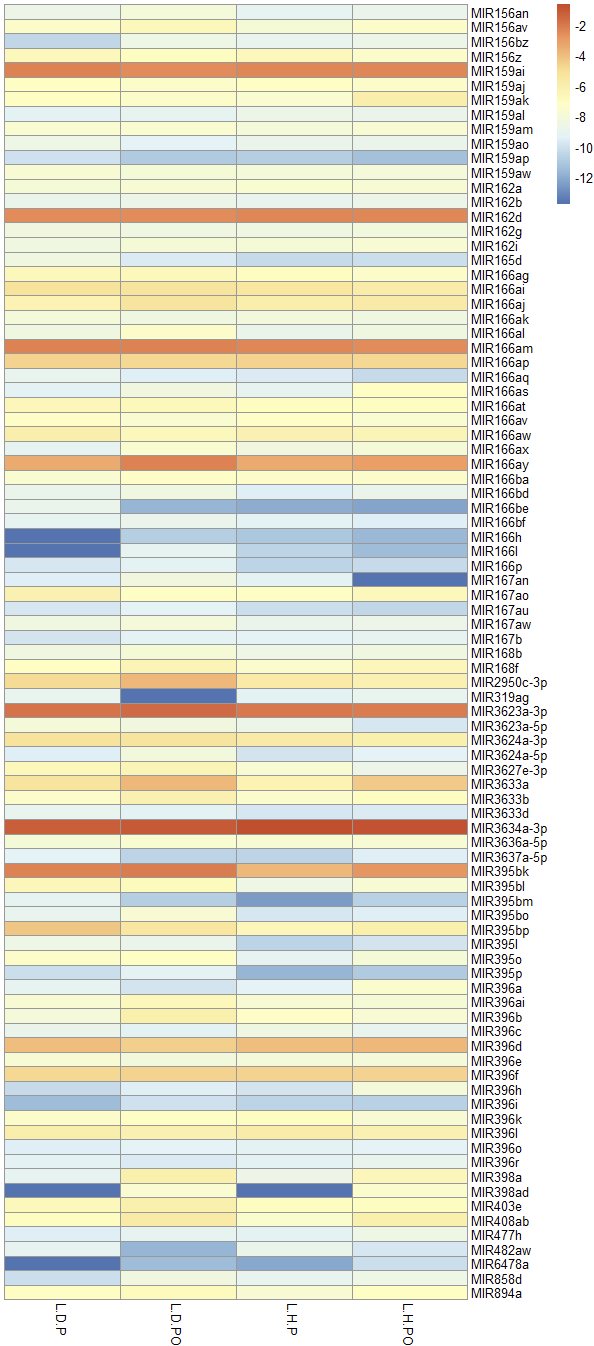
Figure S4: miRNA abundance heatmaps**

Heatmaps showing normalized abundances of miRNAs which passed initial cutoffs (at least one count in a minimum of six different samples) in leaves (left) and berries (right). The numeric scale is derived from the normalization coefficients generated in ‘edgeR,’ such that the further a number is from zero, the lower the relative expression.
